# Supplementary material for: Balance correlations, agentic zeros, and networks: The structure of 192 years of war and peace
Source: PLoS One. 2024 Dec 20;19(12):e0315088. doi: 10.1371/journal.pone.0315088 (PMC11661612; doi:10.1371/journal.pone.0315088)
Supplement: S2 File — It is advised to first download the data and save them in the same folder from which the notebook is running under the names used in the notebook. In addition, it contains the code that was used in pre-processing the data and generating the yearly networks on the three different relations. By providing this notebook, we enable readers to inspect the code and understand the underlying methodology and replicate the data generation process for their own research. The required libraries are pandas and numpy. (PDF) [file pone.0315088.s002.pdf]

CountryRelationGenerator

December 7, 2024

[ ]:
```python
# import packages
import pandas as pd
import numpy as np
```

0.0.1 Load country code

[ ]:
```python
# Data available at https://correlatesofwar.org/data-sets/cow-country-codes-2/
country_code = pd.read_csv('COW country codes.csv').
↪drop_duplicates(ignore_index=True)
country_code
```

0.0.2 Country Start & End Date

[3]:
```python
# Data available at https://correlatesofwar.org/data-sets/
↪state-system-membership/
country_start_end = pd.read_csv("states2016 - list of countries start and end␣
↪dates.csv")
country_start_end
```

[3]:
```
    stateabb  ccode     statenme  styear  stmonth  stday  endyear  endmonth  \
0        AFG    700  Afghanistan    1919        8      8     2016        12
1        ALB    339      Albania    1914        1      1     1939         4
2        ALB    339      Albania    1944       11     17     2016        12
3        ALG    615      Algeria    1962        7      5     2016        12
4        AND    232      Andorra    1993        7     28     2016        12
..       …      …          …      …       …      …       …        …
238      YUG    345   Yugoslavia    1878        7     13     1941         4
239      YUG    345   Yugoslavia    1944       10     20     2016        12
240      ZAM    551       Zambia    1964       10     24     2016        12
241      ZAN    511     Zanzibar    1963       12     19     1964         4
242      ZIM    552     Zimbabwe    1965       11     11     2016        12

     endday  version
0        31     2016
1         7     2016
2        31     2016
3        31     2016
```

```
4          31      2016
..        …       …
238        20      2016
239        31      2016
240        31      2016
241        26      2016
242        31      2016

[243 rows x 10 columns]
```

[25]:
```
country_start_end[country_start_end['statenme'] == 'China']
```

[25]:
```
    ccode statenme  year
41    710    China  1860
41    710    China  1861
41    710    China  1862
41    710    China  1863
41    710    China  1864
..    …        …    …
41    710    China  2012
41    710    China  2013
41    710    China  2014
41    710    China  2015
41    710    China  2016

[157 rows x 3 columns]
```

[4]:
```
# an example
country_start_end[country_start_end['ccode'] == 40]
```

[4]:
```
   stateabb  ccode statenme  styear  stmonth  stday  endyear  endmonth  \
47      CUB     40     Cuba    1902        5     20     1906         9
48      CUB     40     Cuba    1909        1     23     2016        12

    endday  version
47      25     2016
48      31     2016
```

[5]:
```
country_start_end['year'] = country_start_end.apply(lambda x :␣
 ↪list(range(x['styear'],x['endyear']+1)), axis=1)
country_start_end = country_start_end[['ccode', 'statenme', 'year']].
 ↪explode('year')
#country_start_end = country_start_end.explode('year_range')
country_start_end
```

[5]:
```
     ccode     statenme  year
0      700  Afghanistan  1919
```

```
0      700  Afghanistan  1920
0      700  Afghanistan  1921
0      700  Afghanistan  1922
0      700  Afghanistan  1923
..     …            …     …
242    552     Zimbabwe  2012
242    552     Zimbabwe  2013
242    552     Zimbabwe  2014
242    552     Zimbabwe  2015
242    552     Zimbabwe  2016

[15951 rows x 3 columns]
```

0.0.3 Negative Relation (war)

[6]:
```python
# Data available at https://correlatesofwar.org/wp-content/uploads/
↪Dyadic-Interstate-War-Dataset.zip
# read data
war = pd.read_csv("Inter-StateWarData_v4.0.csv")
war
```

[6]:
```
     WarNum                WarName  WarType  ccode  \
0         1      Franco-Spanish War        1    230
1         1      Franco-Spanish War        1    220
2         4     First Russo-Turkish        1    640
3         4     First Russo-Turkish        1    365
4         7        Mexican-American        1     70
..      …                       …      …     …
332     225  Invasion of Afghanistan        1    700
333     227        Invasion of Iraq        1    900
334     227        Invasion of Iraq        1    200
335     227        Invasion of Iraq        1      2
336     227        Invasion of Iraq        1    645

                    StateName  Side  StartMonth1  StartDay1  StartYear1  \
0                       Spain     2            4          7        1823
1                      France     1            4          7        1823
2              Ottoman Empire     2            4         26        1828
3                      Russia     1            4         26        1828
4                      Mexico     2            4         25        1846
..                        …    …          …        …          …
332               Afghanistan     2           10          7        2001
333                 Australia     1            3         19        2003
334            United Kingdom     1            3         19        2003
335  United States of America     1            3         19        2003
336                      Iraq     2            3         19        2003
```

```
     EndMonth1  …  EndMonth2  EndDay2  EndYear2  TransFrom  WhereFought  \
0           11  …         -8       -8        -8        503            2
1           11  …         -8       -8        -8        503            2
2            9  …         -8       -8        -8        506           11
3            9  …         -8       -8        -8        506           11
4            9  …         -8       -8        -8         -8            1
..         …  …        …      …       …       …          …
332         12  …         -8       -8        -8        851            7
333          5  …         -8       -8        -8         -8            6
334          5  …         -8       -8        -8         -8            6
335          5  …         -8       -8        -8         -8            6
336          5  …         -8       -8        -8         -8            6

     Initiator  Outcome  TransTo BatDeath  Version
0            2        2       -8      600        4
1            1        1       -8      400        4
2            2        2       -8    80000        4
3            1        1       -8    50000        4
4            2        2       -8     6000        4
..         …      …      …      …      …
332          2        4      481     4000        4
333          1        4      482        0        4
334          1        4      482       33        4
335          1        4      482      140        4
336          2        4      482     7000        4

[337 rows x 25 columns]
```

[7]:
```python
# pair two sides
side1 = war[war['Side'] == 1][['WarName', 'StateName', 'ccode', 'StartYear1',␣
 ↪'EndYear1']]
side2 = war[war['Side'] == 2][['WarName', 'StateName', 'ccode', 'StartYear1',␣
 ↪'EndYear1']]
war_pairs1 = side1.merge(side2, how='outer', on='WarName')
war_pairs2 = side2.merge(side1, how='outer', on='WarName')
war_pairs = pd.concat([war_pairs1, war_pairs2], ignore_index=True)
war_pairs
```

[7]:
```
                        WarName               StateName_x  ccode_x  StartYear1_x  \
0          Franco-Spanish War                    France      220          1823
1         First Russo-Turkish                    Russia      365          1828
2            Mexican-American  United States of America        2          1846
3            Austro-Sardinian                   Austria      300          1848
4            Austro-Sardinian                   Austria      300          1848
..                        …                       …      …           …
879  Invasion of Afghanistan               Afghanistan      700          2001
880  Invasion of Afghanistan               Afghanistan      700          2001
```

```
881          Invasion of Iraq                            Iraq      645            2003
882          Invasion of Iraq                            Iraq      645            2003
883          Invasion of Iraq                            Iraq      645            2003

     EndYear1_x               StateName_y  ccode_y  StartYear1_y  EndYear1_y
0          1823                     Spain      230          1823        1823
1          1829           Ottoman Empire       640          1828        1829
2          1847                    Mexico       70          1846        1847
3          1848                   Tuscany      337          1848        1848
4          1848                     Italy      325          1848        1848
..          ...                       ...      ...           ...         ...
879        2001  United States of America        2          2001        2001
880        2001                 Australia      900          2001        2001
881        2003                 Australia      900          2003        2003
882        2003            United Kingdom      200          2003        2003
883        2003  United States of America        2          2003        2003

[884 rows x 9 columns]
```

[8]:
```
war_pairs[war_pairs['WarName'] == 'Franco-Spanish War']
```

[8]:
```
                WarName StateName_x  ccode_x  StartYear1_x  EndYear1_x  \
0    Franco-Spanish War      France      220          1823        1823
442  Franco-Spanish War       Spain      230          1823        1823

    StateName_y  ccode_y  StartYear1_y  EndYear1_y
0         Spain      230          1823        1823
442      France      220          1823        1823
```

[9]:
```
# extract overlapping war years
max_start = war_pairs[['StartYear1_x', 'StartYear1_y']].max(axis=1)
min_end = war_pairs[['EndYear1_x', 'EndYear1_y']].min(axis=1)
war_pairs['overlap_years'] = [[]] * len(war_pairs)
for i in range(len(war_pairs)):
    war_pairs['overlap_years'][i] = np.arange(max_start[i], min_end[i] + 1)
war_pairs
```

```
C:\Users\slyuc\AppData\Local\Temp/ipykernel_11940/1377767396.py:6:
SettingWithCopyWarning:
A value is trying to be set on a copy of a slice from a DataFrame

See the caveats in the documentation: https://pandas.pydata.org/pandas-
docs/stable/user_guide/indexing.html#returning-a-view-versus-a-copy
  war_pairs['overlap_years'][i] = np.arange(max_start[i], min_end[i] + 1)
```

[9]:
```
                     WarName              StateName_x  ccode_x  StartYear1_x  \
0         Franco-Spanish War                   France      220          1823
1        First Russo-Turkish                   Russia      365          1828
```

```
2          Mexican-American  United States of America       2          1846
3          Austro-Sardinian                   Austria     300          1848
4          Austro-Sardinian                   Austria     300          1848
..                      …                        …       …             …
879   Invasion of Afghanistan             Afghanistan     700          2001
880   Invasion of Afghanistan             Afghanistan     700          2001
881          Invasion of Iraq                    Iraq     645          2003
882          Invasion of Iraq                    Iraq     645          2003
883          Invasion of Iraq                    Iraq     645          2003

     EndYear1_x               StateName_y  ccode_y  StartYear1_y  EndYear1_y  \
0          1823                     Spain      230          1823        1823
1          1829            Ottoman Empire      640          1828        1829
2          1847                    Mexico       70          1846        1847
3          1848                   Tuscany      337          1848        1848
4          1848                     Italy      325          1848        1848
..          …                        …       …             …           …
879        2001  United States of America        2          2001        2001
880        2001                 Australia      900          2001        2001
881        2003                 Australia      900          2003        2003
882        2003            United Kingdom      200          2003        2003
883        2003  United States of America        2          2003        2003

    overlap_years
0          [1823]
1    [1828, 1829]
2    [1846, 1847]
3          [1848]
4          [1848]
..            …
879        [2001]
880        [2001]
881        [2003]
882        [2003]
883        [2003]

[884 rows x 10 columns]
```

[10]:
```python
war_pairs = war_pairs.explode('overlap_years')
war_pairs
```

[10]:
```
                 WarName               StateName_x  ccode_x  StartYear1_x  \
0      Franco-Spanish War                    France      220          1823
1     First Russo-Turkish                    Russia      365          1828
1     First Russo-Turkish                    Russia      365          1828
2        Mexican-American  United States of America        2          1846
2        Mexican-American  United States of America        2          1846
```

```
..                         …                        …     …          …
879  Invasion of Afghanistan              Afghanistan     700        2001
880  Invasion of Afghanistan              Afghanistan     700        2001
881         Invasion of Iraq                     Iraq     645        2003
882         Invasion of Iraq                     Iraq     645        2003
883         Invasion of Iraq                     Iraq     645        2003

     EndYear1_x               StateName_y  ccode_y  StartYear1_y  EndYear1_y  \
0          1823                     Spain      230          1823        1823
1          1829            Ottoman Empire      640          1828        1829
1          1829            Ottoman Empire      640          1828        1829
2          1847                    Mexico       70          1846        1847
2          1847                    Mexico       70          1846        1847
..          …                        …       …            …          …
879        2001  United States of America        2          2001        2001
880        2001                 Australia      900          2001        2001
881        2003                 Australia      900          2003        2003
882        2003            United Kingdom      200          2003        2003
883        2003  United States of America        2          2003        2003

    overlap_years
0            1823
1            1828
1            1829
2            1846
2            1847
..            …
879          2001
880          2001
881          2003
882          2003
883          2003

[2046 rows x 10 columns]
```

[11]:
```
negative = war_pairs.dropna()
negative = negative[['StateName_x', 'StateName_y', 'ccode_x', 'ccode_y',␣
 ↪'overlap_years']]
negative['Negative Relation'] = True
negative = negative.rename(columns={'overlap_years': 'Year'})
negative
```

[11]:
```
                  StateName_x            StateName_y  ccode_x  ccode_y  \
0                      France                  Spain      220      230
1                      Russia         Ottoman Empire      365      640
1                      Russia         Ottoman Empire      365      640
2    United States of America                 Mexico        2       70
```

```
2    United States of America                        Mexico        2       70
..                          …                           …       …       …
879                 Afghanistan  United States of America      700        2
880                 Afghanistan                 Australia      700      900
881                        Iraq                 Australia      645      900
882                        Iraq            United Kingdom      645      200
883                        Iraq  United States of America      645        2

     Year  Negative Relation
0    1823               True
1    1828               True
1    1829               True
2    1846               True
2    1847               True
..    …                  …
879  2001               True
880  2001               True
881  2003               True
882  2003               True
883  2003               True

[1968 rows x 6 columns]
```

0.0.4 Positive Relation

Defense Cooperation

[12]:
```python
#Data available from https://correlatesofwar.org/data-sets/
↪defense-cooperation-agreement-dataset/
defense_cooperation = pd.read_csv('./defense_cooperation/DCAD-v1.0-dyadic.csv')
defense_cooperation = defense_cooperation[['ccode1', 'abbrev1', 'ccode2',␣
↪'abbrev2', 'year']]
defense_cooperation
```

[12]:
```
        ccode1 abbrev1  ccode2 abbrev2  year
0            2     USA      20     CAN  1980
1            2     USA      20     CAN  1981
2            2     USA      20     CAN  1982
3            2     USA      20     CAN  1983
4            2     USA      20     CAN  1984
…          …      …      …      …    …
994493     990     WSM     987     FSM  2006
994494     990     WSM     987     FSM  2007
994495     990     WSM     987     FSM  2008
994496     990     WSM     987     FSM  2009
994497     990     WSM     987     FSM  2010

[994498 rows x 5 columns]
```

Intergovernmental Organizations

[13]:
```
#Data available from https://correlatesofwar.org/wp-content/uploads/
↪dyadic_formatv3.zip
intergovernmental_organizations = pd.read_csv('dyadic_formatv3.csv')
intergovernmental_organizations = intergovernmental_organizations[['ccode1',␣
↪'country1', 'ccode2', 'country2', 'year']]
intergovernmental_organizations
```

[13]:
```
        ccode1 country1  ccode2 country2  year
0            2      USA      20      CAN  1920
1            2      USA      20      CAN  1921
2            2      USA      20      CAN  1922
3            2      USA      20      CAN  1923
4            2      USA      20      CAN  1924
…          …       …      …       …   …
917690     987      FSM     990      WSM  2010
917691     987      FSM     990      WSM  2011
917692     987      FSM     990      WSM  2012
917693     987      FSM     990      WSM  2013
917694     987      FSM     990      WSM  2014

[917695 rows x 5 columns]
```

Formal Alliances

[14]:
```
#Data available from https://correlatesofwar.org/wp-content/uploads/version4.
↪1_csv.zip
formal_alliances = pd.read_csv('./alliance/alliance_v4.1_by_dyad_yearly.csv')
formal_alliances
```

[14]:
```
       version4id  ccode1               state_name1  ccode2 state_name2  \
0               1     200            United Kingdom     235    Portugal
1               1     200            United Kingdom     235    Portugal
2               1     200            United Kingdom     235    Portugal
3               1     200            United Kingdom     235    Portugal
4               1     200            United Kingdom     235    Portugal
…             …      …                      …     …       …
74124         414       2  United States of America     666      Israel
74125         414       2  United States of America     666      Israel
74126         414       2  United States of America     666      Israel
74127         414       2  United States of America     666      Israel
74128         414       2  United States of America     666      Israel

       dyad_st_day  dyad_st_month  dyad_st_year  dyad_end_day  dyad_end_month  \
0                1              1          1816           NaN             NaN
1                1              1          1816           NaN             NaN
2                1              1          1816           NaN             NaN
3                1              1          1816           NaN             NaN
```

```
4                1              1          1816           NaN             NaN
…             …             …            …            …              …
74124           30             11          1987          26.0            12.0
74125           30             11          1988          26.0            12.0
74126           30             11          1989          26.0            12.0
74127           30             11          1990          26.0            12.0
74128           30             11          1991          26.0            12.0

       dyad_end_year  left_censor  right_censor  defense  neutrality  \
0               2012            1             1        1           0
1               2012            1             1        1           0
2               2012            1             1        1           0
3               2012            1             1        1           0
4               2012            1             1        1           0
…                …            …            …       …          …
74124           1991            0             0        0           0
74125           1991            0             0        0           0
74126           1991            0             0        0           0
74127           1991            0             0        0           0
74128           1991            0             0        0           0

       nonaggression  entente  year  version
0                1.0      0.0  1816      4.1
1                1.0      0.0  1817      4.1
2                1.0      0.0  1818      4.1
3                1.0      0.0  1819      4.1
4                1.0      0.0  1820      4.1
…                 …     …    …      …
74124            0.0      1.0     0      4.1
74125            0.0      1.0     0      4.1
74126            0.0      1.0     0      4.1
74127            0.0      1.0     0      4.1
74128            0.0      1.0     0      4.1

[74129 rows x 19 columns]
```

[15]:
```python
def find_year(x):
    if x['year'] == 0:
        x['year'] = x['dyad_st_year']
        return x
    else:
        return x
formal_alliances = formal_alliances.apply(find_year, axis=1)
formal_alliances
```

[15]:
```
       version4id  ccode1            state_name1  ccode2 state_name2  \
0               1     200         United Kingdom     235    Portugal
```

```
1              1    200           United Kingdom    235    Portugal
2              1    200           United Kingdom    235    Portugal
3              1    200           United Kingdom    235    Portugal
4              1    200           United Kingdom    235    Portugal
…            …    …                         …      …       …
74124        414      2  United States of America    666      Israel
74125        414      2  United States of America    666      Israel
74126        414      2  United States of America    666      Israel
74127        414      2  United States of America    666      Israel
74128        414      2  United States of America    666      Israel

       dyad_st_day  dyad_st_month  dyad_st_year  dyad_end_day  dyad_end_month  \
0                1              1          1816           NaN             NaN
1                1              1          1816           NaN             NaN
2                1              1          1816           NaN             NaN
3                1              1          1816           NaN             NaN
4                1              1          1816           NaN             NaN
…              …              …            …             …               …
74124           30             11          1987          26.0            12.0
74125           30             11          1988          26.0            12.0
74126           30             11          1989          26.0            12.0
74127           30             11          1990          26.0            12.0
74128           30             11          1991          26.0            12.0

       dyad_end_year  left_censor  right_censor  defense  neutrality  \
0               2012            1             1        1           0
1               2012            1             1        1           0
2               2012            1             1        1           0
3               2012            1             1        1           0
4               2012            1             1        1           0
…                …            …             …       …          …
74124           1991            0             0        0           0
74125           1991            0             0        0           0
74126           1991            0             0        0           0
74127           1991            0             0        0           0
74128           1991            0             0        0           0

       nonaggression  entente  year  version
0                1.0      0.0  1816      4.1
1                1.0      0.0  1817      4.1
2                1.0      0.0  1818      4.1
3                1.0      0.0  1819      4.1
4                1.0      0.0  1820      4.1
…                …      …    …       …
74124            0.0      1.0  1987      4.1
74125            0.0      1.0  1988      4.1
74126            0.0      1.0  1989      4.1
```

```
74127             0.0      1.0  1990      4.1
74128             0.0      1.0  1991      4.1

[74129 rows x 19 columns]
```

[16]:
```
formal_alliances =␣
 ↪formal_alliances[['ccode1','state_name1','ccode2','state_name2', 'year']]
formal_alliances
```

[16]:
```
       ccode1              state_name1  ccode2 state_name2  year
0         200           United Kingdom     235    Portugal  1816
1         200           United Kingdom     235    Portugal  1817
2         200           United Kingdom     235    Portugal  1818
3         200           United Kingdom     235    Portugal  1819
4         200           United Kingdom     235    Portugal  1820
…       …                       …     …          …   …
74124       2  United States of America     666      Israel  1987
74125       2  United States of America     666      Israel  1988
74126       2  United States of America     666      Israel  1989
74127       2  United States of America     666      Israel  1990
74128       2  United States of America     666      Israel  1991

[74129 rows x 5 columns]
```

0.0.5 Clean and Combine

[17]:
```
negative = negative.rename(columns={'ccode_x': 'ccode1', 'ccode_y': 'ccode2',␣
 ↪'Year': 'year'})
defense_cooperation['Defense Cooperation'] = True
formal_alliances['Formal Alliances'] = True
intergovernmental_organizations['Intergovernmental Organizations'] = True
```

```
C:\Users\slyuc\AppData\Local\Temp/ipykernel_11940/3871866741.py:3:
SettingWithCopyWarning:
A value is trying to be set on a copy of a slice from a DataFrame.
Try using .loc[row_indexer,col_indexer] = value instead

See the caveats in the documentation: https://pandas.pydata.org/pandas-
docs/stable/user_guide/indexing.html#returning-a-view-versus-a-copy
  formal_alliances['Formal Alliances'] = True
```

[18]:
```
temp = negative.merge(formal_alliances, how='outer', on=['ccode1', 'ccode2',␣
 ↪'year'])
temp = temp.merge(defense_cooperation, how='outer', on=['ccode1', 'ccode2',␣
 ↪'year'])
combined = temp.merge(intergovernmental_organizations, how='outer',␣
 ↪on=['ccode1', 'ccode2', 'year'])
```

```
combined = combined[['ccode1', 'ccode2', 'year', 'Negative Relation', 'Formal␣
↪Alliances', 'Defense Cooperation', 'Intergovernmental Organizations']]
combined
```

[18]:

```
         ccode1  ccode2    year Negative Relation Formal Alliances  \
0           220     230  1823.0              True              NaN
1           365     640  1829.0              True              NaN
2           365     640  1829.0              True              NaN
3             2      70  1846.0              True              NaN
4             2      70  1847.0              True              NaN
…           …       …       …               …                …
1430848     986     990  2014.0               NaN              NaN
1430849     987     990  2011.0               NaN              NaN
1430850     987     990  2012.0               NaN              NaN
1430851     987     990  2013.0               NaN              NaN
1430852     987     990  2014.0               NaN              NaN

        Defense Cooperation Intergovernmental Organizations
0                       NaN                            True
1                       NaN                            True
2                       NaN                            True
3                       NaN                            True
4                       NaN                            True
…                       …                              …
1430848                 NaN                            True
1430849                 NaN                            True
1430850                 NaN                            True
1430851                 NaN                            True
1430852                 NaN                            True

[1430853 rows x 7 columns]
```

[19]:

```
combined = combined.fillna(False)
combined['year'] = combined['year'].astype(int)
combined['Formal Alliances'] = combined['Formal Alliances'].replace(True,␣
↪'Formal Alliances')
combined['Formal Alliances'] = combined['Formal Alliances'].replace(False, '')
combined['Defense Cooperation'] = combined['Defense Cooperation'].replace(True,␣
↪'Defense Cooperation')
combined['Defense Cooperation'] = combined['Defense Cooperation'].
↪replace(False, '')
combined['Intergovernmental Organizations'] = combined['Intergovernmental␣
↪Organizations'].replace(True, 'Intergovernmental Organizations')
combined['Intergovernmental Organizations'] = combined['Intergovernmental␣
↪Organizations'].replace(False, '')
```

```
combined['Positive Relation'] = combined['Formal Alliances'] + ',' +␣
↪combined['Defense Cooperation'] + ',' + combined['Intergovernmental␣
↪Organizations']
combined['Positive Relation'] = combined['Positive Relation'].str.split(',').
↪apply(lambda row: [x for x in row if x])
combined = combined[['ccode1', 'ccode2', 'year', 'Negative Relation', 'Positive␣
↪Relation']]
combined
```

[19]:

```
         ccode1  ccode2  year  Negative Relation  \
0           220     230  1823               True
1           365     640  1828               True
2           365     640  1829               True
3             2      70  1846               True
4             2      70  1847               True
…           …     …   …                 …
1430848     986     990  2014              False
1430849     987     990  2011              False
1430850     987     990  2012              False
1430851     987     990  2013              False
1430852     987     990  2014              False

                        Positive Relation
0        [Intergovernmental Organizations]
1        [Intergovernmental Organizations]
2        [Intergovernmental Organizations]
3        [Intergovernmental Organizations]
4        [Intergovernmental Organizations]
…                                     …
1430848  [Intergovernmental Organizations]
1430849  [Intergovernmental Organizations]
1430850  [Intergovernmental Organizations]
1430851  [Intergovernmental Organizations]
1430852  [Intergovernmental Organizations]

[1430853 rows x 5 columns]
```

[20]:

```
country_code_mapper = dict(zip(country_code.CCode, country_code.StateNme))
combined['country1'] = combined['ccode1'].map(country_code_mapper)
combined['country2'] = combined['ccode2'].map(country_code_mapper)
combined
```

[20]:

```
         ccode1  ccode2  year  Negative Relation  \
0           220     230  1823               True
1           365     640  1828               True
2           365     640  1829               True
3             2      70  1846               True
```

```
4              2      70  1847               True
…          …     …    …                 …
1430848      986     990  2014              False
1430849      987     990  2011              False
1430850      987     990  2012              False
1430851      987     990  2013              False
1430852      987     990  2014              False

                        Positive Relation                        country1  \
0        [Intergovernmental Organizations]                          France
1        [Intergovernmental Organizations]                          Russia
2        [Intergovernmental Organizations]                          Russia
3        [Intergovernmental Organizations]        United States of America
4        [Intergovernmental Organizations]        United States of America
…                                        …                               …
1430848  [Intergovernmental Organizations]                           Palau
1430849  [Intergovernmental Organizations]  Federated States of Micronesia
1430850  [Intergovernmental Organizations]  Federated States of Micronesia
1430851  [Intergovernmental Organizations]  Federated States of Micronesia
1430852  [Intergovernmental Organizations]  Federated States of Micronesia

        country2
0          Spain
1         Turkey
2         Turkey
3         Mexico
4         Mexico
…            …
1430848    Samoa
1430849    Samoa
1430850    Samoa
1430851    Samoa
1430852    Samoa

[1430853 rows x 7 columns]
```

[21]:
```
country_start_end
```

[21]:
```
     ccode     statenme  year
0      700  Afghanistan  1919
0      700  Afghanistan  1920
0      700  Afghanistan  1921
0      700  Afghanistan  1922
0      700  Afghanistan  1923
..     …          …    …
242    552     Zimbabwe  2012
242    552     Zimbabwe  2013
```

```
242     552      Zimbabwe   2014
242     552      Zimbabwe   2015
242     552      Zimbabwe   2016

[15951 rows x 3 columns]
```

[22]:
```
combined = country_start_end.merge(combined, how='left', left_on=['ccode',␣
↪'year'], right_on=['ccode1', 'year'])
combined = combined[['statenme', 'ccode', 'country2', 'ccode2', 'year',␣
↪'Negative Relation', 'Positive Relation']]
combined
```

[22]:
```
            statenme  ccode                        country2  ccode2  year  \
0        Afghanistan    700                           China   710.0  1919
1        Afghanistan    700                           Japan   740.0  1919
2        Afghanistan    700                        Thailand   800.0  1919
3        Afghanistan    700                           China   710.0  1920
4        Afghanistan    700                           Japan   740.0  1920
…                …    …                             …     …   …
1431244     Zimbabwe    552                           Palau   986.0  2014
1431245     Zimbabwe    552  Federated States of Micronesia   987.0  2014
1431246     Zimbabwe    552                           Samoa   990.0  2014
1431247     Zimbabwe    552                             NaN     NaN  2015
1431248     Zimbabwe    552                             NaN     NaN  2016

        Negative Relation                  Positive Relation
0                   False  [Intergovernmental Organizations]
1                   False  [Intergovernmental Organizations]
2                   False  [Intergovernmental Organizations]
3                   False  [Intergovernmental Organizations]
4                   False  [Intergovernmental Organizations]
…                       …                                  …
1431244             False  [Intergovernmental Organizations]
1431245             False  [Intergovernmental Organizations]
1431246             False  [Intergovernmental Organizations]
1431247               NaN                                NaN
1431248               NaN                                NaN

[1431249 rows x 7 columns]
```

[23]:
```
# final clean up

#combined = combined.dropna()
#combined['ccode2'] = combined['ccode2'].astype(int)

#combined
```

0.0.6 Output

[24]:
```
# COW War Data: 1816 - 2007 (v4.0)
# formal alliances: 1816 - 2012
# intergovernmental organizations: 1815 - 2014
# defense cooperation: 1980-2010
combined.to_csv('country_relation_dyadic.csv', index=False)
```

[ ]:
```

```
