# Supplementary material for: Balance correlations, agentic zeros, and networks: The structure of 192 years of war and peace
Source: PLoS One. 2024 Dec 20;19(12):e0315088. doi: 10.1371/journal.pone.0315088 (PMC11661612; doi:10.1371/journal.pone.0315088)
Supplement: S1 Appendix — (PDF) [file pone.0315088.s004.pdf]

S1 Appendix. Proof of theorem 2.1 The claim in Theorem 2.1 is based on the fact that if the indices of the two variables in the balance correlation are permuted in the same way, the correlation does not change due to associative and commutative properties of the addition of the cross-products in the correlation function. Transposing matrices is one unique permutation of the elements in a matrix.

Definition 1. *Let $\mathfrak{M} = \{M_1, ..., M_m\}$ be the set of m square matrices. All matrices M_q are non-empty with zero diagonals and are binary such that each element $M_{q_{ij}} \in \{0, 1\}$. Further, $m \geq 2$, and $\sum_{q=1}^{m} M_q = \mathfrak{L}$, is the matrix representation of the complete graph. Also, all matrices in $\mathfrak{M}$ have identical and identically ordered row and column indices, $i, j \in \{1, ..., n\}$ for the n number of nodes.*

Definition 2. *Let $\mathfrak{M}^{'} = \{M_1^{'}, ..., M_m^{'}\}$ be the set of transposes of matrices in $\mathfrak{M}$. A **nugget** is defined to be the element-wise product of any matrix in $\mathfrak{M}$ with any matrix in $\mathfrak{M}^{'}$. The nugget set $\mathfrak{U} = \mathfrak{M} \times \mathfrak{M}^{'} = \{U_1, ..., U_g\}$ and contains all possible g nuggets. A nugget thus has the same indices of the matrices in $\mathfrak{M}$ and is also binary. There are $g = m^2$ possible nuggets.*

Lemma 1. *In the nugget set $\mathfrak{U}$ consisting of g nuggets, there will be exactly m symmetric nuggets, and exactly $m^2 - m$ asymmetric nuggets.*

Proof of lemma 1. Note that $M_{q_{ji}} = M_{q_{ij}}^{'}$. By contradiction, assume for any ij-th element in a nugget, the element-wise product of matrices, $M_{r_{ij}} \times M_{s_{ji}} = 1$ and $M_{r_{ji}} \times M_{s_{ij}} = 1$. Since, $M_{q_{ij}} \in \{0, 1\}$, this implies $M_{r_{ij}} = M_{s_{ji}} = 1$ and $M_{r_{ji}} = M_{s_{ij}} = 1$, so $M_{r_{ij}} + M_{s_{ij}} = 2$. However, if $r \neq s$ this contradicts that the sum over all matrices results in a matrix $\mathfrak{L}$ (see definition 1). If and only if $r = s$ the assumption holds, as the summation visits each matrix q in $\mathfrak{M}$ only once, which implies there are exactly m symmetric nuggets; those of a matrix and it's transpose. □

Definition 3. *Let a balance correlation be the Pearson correlation between elements of a nugget U_k and the elements of the inner product of two nuggets $U_l U_m$, ignoring the diagonals, i.e.,*

$$\rho(U_k, U_l U_m) = \frac{\operatorname{cov}(U_k, U_l U_m)}{\sqrt{\operatorname{var}(U_k) \operatorname{var}(U_l U_m)}} \tag{1}$$

*Now, $\mathfrak{B}$, is the set containing all **possibly unique** balance correlations that can be derived from the g nuggets in any set $\mathfrak{U}$. The specific order of the nuggets (and hence the matrices they are derived from) in a balance correlation, reveal a statement about the association between a 2-path and tie in bi-directional relations.*

So, $\rho(U_k, U_l U_m)$ is a quantification of the statement about an association between ties in U_k and 2-paths in $U_l U_M$. This quantification differs from $\rho(U_k, U_m U_l)$ or $\rho(U_l, U_k U_m)$, which quantify statements of association between ties in U_k and $U_m U_l$, and, U_l and $U_k U_m$, respectively.

Now, since the bi-variate Pearson correlation in Eq 1 is defined in terms of variances and covariance, and those statistics are permutation invariant, i.e. for any permutation function π_p we have

$$\rho(U_k, U_l U_m) = \rho(\pi_p(U_k), \pi_p(U_l)\pi_p(U_m)). \tag{2}$$

Given that there are m^2 nuggets, and that balance correlation considers 3 nuggets, there are m^6 possible balance correlations. However these are not all unique, because transposition is a permutation and by definition 2, nuggets include transposed matrices.

Nuggets, as Hadamard products that maintain associativity, imply that the order of matrices within a nugget is not important ($M_r \times M_s^{'} = M_s^{'} \times M_r$). However, it is relevant which matrix

in a nugget is transposed in revealing the underlying theoretical statement. Furthermore, the second variable, U_lU_m, is a matrix inner product. It is well-known that taking the transpose of an inner product, transposes the matrices and reverses their order. There are three situations in which transposing either or both variables in ρ changes the formulation of the underlying statement or in other words demonstrate equivalence between two underlying statements under all possible instantiates. Only, when both U_k and U_lU_m are symmetric by construction ρ quantifies a *sole* statement. In the other cases we speak of *twin* statements.

Proof of theorem 2.1. Assume nuggets $\{U_k, U_l, U_m\} \in \mathfrak{U}$ and define $\{U_k = U_q^{'}, U_l = U_r^{'}, U_m = U_s^{'}\}$ which implies $\{U_q, U_r, U_s\} \in \mathfrak{U}$. Since, transposing is a permutation, by Eq 2 we have

$$\rho(U_k, U_lU_m) = \rho(U_k^{'}, U_m^{'}U_l^{'}),$$

which by substitution gives $\rho(U_k, U_lU_m) = \rho(U_q, U_sU_r)$. If and only if, $U_k = U_k^{'} \neq U_q$, and, $U_lU_m = U_m^{'}U_l^{'} \neq U_sU_r$ no other quantification exists that will always produce the same quantity. By the fact that there exists one and only one possible transpose of each nugget, in all other cases there exists exactly one other configuration of distinguishable nuggets that will lead to the same quantification, where $U_k = U_k^{'} = U_q$, and, $U_lU_m = U_m^{'}U_l^{'} = U_sU_r$. □

It follows that quantifying a sole statement requires a symmetric nugget U_k, which implies by lemma 1 the matrices in U_k are M_t and $M_t^{'}$, as well as that $U_l = U_m^{'}$, which implies $U_l = M_vM_w^{'}$ and $U_m = M_wM_v^{'}$. In those cases, one and only one configuration of nuggets (and matrices) exists, since transposing equals the trivial identity permutation. If $U_k = U_q^{'}$ for any $U_k = M_t \times M_s^{'}$ and $U_q = M_s \times M_t^{'}$ will contain the same set of values, but in a different order. Similarly, in any U_lU_m that has U_sU_r as a distinguishable transpose, all values U_lU_m uniquely exist as transpose in U_sU_r, and vice verse. So for any $U_l = M_vM_w^{'}$ and $U_m = M_xM_y^{'}$ there exists $U_r = M_wM_v^{'}$ and $U_s = M_yM_x^{'}$, such that $\sum_k(M_{v_{ik}}M_{w_{ki}})(M_{x_{kj}}M_{y_{jk}}) = \sum_k(M_{y_{jk}}M_{x_{kj}})(M_{v_{ik}}M_{w_{ki}})\,\forall\,\{i,j,k\}$. Hence, only when either or both variables in ρ have a distinguishable transpose a distinguishable twin statement exists that produces the exact same value for ρ, i.e. showing duality for this subset of balance correlations.

Cardinality

Definition 3 determines that any balance correlation $\rho(U_k, U_lU_m)$ is a bi-variate Pearson correlation. This leads us to consider two conditions, or distinguishing assumptions, as special cases. Although we recognise more complex situations could apply, these two limiting conditions will provide an upper and lower cardinality bound for $\mathfrak{B}$.

Assumption 1. *All matrices in $\mathfrak{M}$ are symmetric.*

This implies that for all nuggets in $\mathfrak{Z} = \{M_r \times M_s^{'} \,|\, r \neq s, M_r \in \mathfrak{M}, M_s^{'} \in \mathfrak{M}^{'}\}$ the variances $\mathrm{var}(Z_h) = 0$. Since the Pearson correlation is defined if and only if $\mathrm{var}(U_k) > 0$ and $\mathrm{var}(U_lU_m) > 0$, the balance correlations that include Z_h are not defined, and cannot be considered. Therefore, under assumption 1 the nugget set cardinality will be $|\mathfrak{U}| = m$, and will only contain symmetric nuggets.

The symmetry of the nuggets implies there are m possible nuggets for the first variable in ρ. Because, there is no restriction on the use of nuggets in any of the 2 positions in the second variable, the number of possible combinations to constitute it, in general, is given by

$$\eta = \left(\!\!\binom{m}{2}\!\!\right) = \frac{(m-1+2)!}{(m-1)!2!}, \tag{3}$$

where m is the number of different nuggets in $\mathfrak{Z}$. It follows that $|\mathfrak{B}| = \eta \cdot m$ is the cardinality of the largest possible set of unique balance correlations.

In the specific case discussed in this paper, where we are limiting ourselves to a set of three symmetric relations (**P**, **N**, and **Z**), $\eta = 6$ and thus the largest possible set of *unique* correlations $= 3 \cdot 6 = 18$. Therefore, of the $m^3 = 27$ possible balance correlations, only 18 of them will be unique, and the remaining nine will be twins, paired with nine elements in this set of 18 unique balance correlations. With nine pairs of twins, that means the remaining nine balance correlations will have no twins.

Assumption 2. *All matrices in* $\mathfrak{M}$ *are asymmetric, nuggets have* $\mathrm{var}(U_k) > 0 \,\forall\, k \in \{1, ..., g\}$, *and the inner product of nuggets have* $\mathrm{var}(U_l U_m) > 0 \,\forall\, l, m \in \{1, ..., g\}$.

The proof of theorem 2.1 provides the conditions under which a specific configuration of nuggets doesn't have a twin. It requires a symmetric nugget in the first variable in ρ, and a symmetric inner product of nuggets in the second variable. The latter condition can only hold when no more than two different matrices constitute the two nuggets. The order of the two matrices do define the specific nuggets, and hence is important. However, these two nuggets also need to be transposes of each other. Given m asymmetric matrices there are exactly m symmetric nuggets, according to the proof of lemma 1. Furthermore, there are exactly $g = m^2$ nuggets, which includes their transposes. So, the total number of possible inner products equals m^4, of which m^2 will be inner products with the transpose. Hence, there are $\alpha = m \cdot m^2 = m^3$ number of configurations of matrices in the variables of ρ that quantify a sole statement. The other possible balance correlations all have exactly one other equal quantification of a twin statement. Therefore,

$$|\mathfrak{B}| = \frac{1}{2}(m^6 - m^3) + m^3 = \frac{1}{2}m^3(m^3 + 1) \tag{4}$$

where m^6 is the number of combinations of all possible nuggets (2 matrices in a nugget) in the three nuggets used in $\rho(U_k, U_l U_m)$. For any set of mixed symmetric and asymmetric matrices the cardinality of $|\mathfrak{B}|$ will be between those in Eq 3 and Eq 4.

The logical entanglement of statements is both interesting and limiting this approach. In fact it can be solved by extending the approach to consider an extra temporal dimension to the data. Note however, that the balanced states all rely on the assumption of reciprocal relations (symmetric nuggets). As such, at any moment in time these states, according to the theory, may be expected to occur more than unbalanced states. This motivates the use of balance correlation as a cross-sectional concept, and to rely on a subset of $|\mathfrak{B}|$, which reflects the statement. Yet, from an exploratory perspective it remains of interest to inspect the values of other measures as this can reveal information about the process of balance formation.
